# The added value of using mutational profiling in addition to cytology in diagnosing aggressive pancreaticobiliary disease: review of clinical cases at a single center

**DOI:** 10.1186/1471-230X-14-135

**Published:** 2014-08-01

**Authors:** Nidhi Malhotra, Sara A Jackson, Lindsay L Freed, Mindi A Styn, Mary K Sidawy, Nadim G Haddad, Sydney D Finkelstein

**Affiliations:** 1 Medstar Georgetown University Hospital, Division of Gastroenterology, Washington, DC, USA; 2 RedPath Integrated Pathology, Inc., Pittsburgh, PA, USA; 3 Department of Pathology, Medstar Georgetown University Hospital, Washington, DC, USA; 4 Department of Pathology, Drexel University, Philadelphia, PA, USA

**Keywords:** Pancreatic cyst, Pancreatic cancer, Molecular analysis

## Abstract

**Background:**

This study aimed to better understand the supporting role that mutational profiling (MP) of DNA from microdissected cytology slides and supernatant specimens may play in the diagnosis of malignancy in fine-needle aspirates (FNA) and biliary brushing specimens from patients with pancreaticobiliary masses.

**Methods:**

Cytology results were examined in a total of 30 patients with associated surgical (10) or clinical (20) outcomes. MP of DNA from microdissected cytology slides and from discarded supernatant fluid was analyzed in 26 patients with atypical, negative or indeterminate cytology.

**Results:**

Cytology correctly diagnosed aggressive disease in 4 patients. Cytological diagnoses for the remaining 26 were as follows: 16 negative (9 false negative), 9 atypical, 1 indeterminate. MP correctly determined aggressive disease in 1 false negative cytology case and confirmed a negative cytology diagnosis in 7 of 7 cases of non-aggressive disease. Of the 9 atypical cytology cases, MP correctly diagnosed 7 as positive and 1 as negative for aggressive disease. One specimen that was indeterminate by cytology was correctly diagnosed as non-aggressive by MP. When first line malignant (positive) cytology results were combined with positive second line MP results, 12/21 cases of aggressive disease were identified, compared to 4/21 cases identified by positive cytology alone.

**Conclusions:**

When first line cytology results were uncertain (atypical), questionable (negative), or not possible (non-diagnostic/indeterminate), MP provided additional information regarding the presence of aggressive disease. When used in conjunction with first line cytology, MP increased detection of aggressive disease without compromising specificity in patients that were difficult to diagnose by cytology alone.

## Background

Pancreaticobiliary cancer is associated with a 5-year survival rate of less than 6%, which has not changed significantly in the past 30 years [1]. Thus, traditional clinical strategies in early diagnosis and patient treatment have not significantly improved patient outcomes. Although recent advancements in imaging techniques have increased the sensitivity for detecting pancreatic and biliary solid and cystic lesions [2,3], the standard method of diagnosis, cytological evaluation of fine needle aspirates (FNA) and biliary brushing specimens, carries a high false negative rate and often yields findings that are inconclusive [4-7].

False negative cytology results are largely due to sampling variability that can result from FNA and biliary brushing procedures, acellularity of the corresponding specimens, and difficulty in characterizing cellular atypia due to the aforementioned reasons. Sites of advanced disease are inadvertently missed and few to no cells are captured due to biological variation within the neoplastic cell population leading to mischaracterization [8,9]. These confounding variables are especially concerning when dealing with progressively smaller lesions that are detected earlier with advanced imaging. Collectively, these limitations make it clear that methods to support and improve the diagnostic process of pancreaticobiliary cancer would greatly enhance the management and care of patients.

Many studies have highlighted the association between cumulative DNA damage and pancreaticobiliary cancers [10-17]. Some studies have also shown that microdissection-based mutational profiling of DNA can be clinically useful in diagnosing these malignancies [10-15]. Such molecular analysis is especially significant when morphologic methods are uncertain or not obtainable [18-22]. Like cytology, the use of mutational profiling has traditionally required cellular material that shows morphological signs of disease in order to extract DNA from microdissected areas of relevant cells. However, pauci- and acellular specimens at times fail to provide sufficient material for cell-based DNA mutational profiling.

Recent studies have shown successful mutational profiling of DNA not only from microdissected cytology slides but also from cell-free supernatant fluid obtained during preparation of the cytology slides [22]. The supernatant fractions of FNA and biliary brushing specimens are an underutilized and often overlooked source of DNA [22,23]. The supernatant of FNA and biliary brushing specimens is typically discarded but contains DNA that can provide molecular information even when the cell count is low or nonexistent [22,23]. The utilization of the cytocentrifugation supernatant can especially be of significant value in those cases where the cellular material is scarce or even absent without compromising the material routinely used for other diagnostic tests. Numerous studies have reported the utility as well as the high quality of the extracellular DNA that surrounds cancer cells, with some studies observing that it may in fact be more representative of a tumor than intracellular DNA obtained via other methods [24-32].

We aimed to better understand the supporting role that mutational profiling of DNA from microdissected cytology slides and supernatant specimens may play in the accurate diagnosis of malignancy in patients with pancreaticobiliary masses. FNA or biliary brushing procedures were performed to collect diagnostic specimens. Patients with associated surgical or clinical follow-up outcomes were examined by cytopathology as well as mutational profiling of DNA from microdissected cytology slides and from supernatant fluid, which is normally otherwise discarded. More specifically, the study aimed to examine the added value of mutational profiling for detecting aggressive disease when first line testing by cytopathology was either negative or insufficient for diagnosis (i.e. atypical or indeterminate) of malignancy.

## Methods

### Study population

This is a retrospective review of patients who presented with a pancreaticobiliary mass found on cross-sectional imaging at our institution during a ten month period from August 2011 to May 2012. All patients underwent FNA of the pancreaticobiliary mass or biliary brushing if the FNA was not possible. The Georgetown University Institutional Review Board granted approval (# Ame1_2011-553) for review of cytology reports and performance of mutational profiling of FNA and brushing specimens from patients at Medstar Georgetown University Hospital. The patient cohort included both cases with unclear cytology results, such as those with negative, atypical or indeterminate (acellular or insufficient cells for cytology diagnosis) results, as well as cases with definitive positive cytology results describing the presence of malignant disease.

Patient outcomes were categorized as “aggressive” based on either surgical pathology or oncology follow-up report indications of adenocarcinoma, cholangiocarcinoma, intraductal tubulo-papillary neoplasm, pancreatic cancer treatment, or death from pancreatic cancer. Patient outcomes were categorized as “non-aggressive” based on at least 3 months of clinical follow-up via cross sectional imaging or repeat endoscopic ultrasound indicating resolution or stable (unchanging or shrinking) pancreaticobiliary mass.

### Cytology

Pancreaticobiliary mass FNA and biliary brushing specimens were fixed, followed by standard cytology slide preparation. The normally discarded cell-free supernatant fluid remaining after centrifugation of the cells during cytology preparation was saved at 4°C for mutational profiling.

Cytology results were categorized as “positive”, “atypical”, “negative” or “indeterminate” based on language abstracted from the cytology report. Any case with a cytology report that clearly noted the presence of adenocarcinoma or malignancy was defined as “positive” for aggressive disease by cytology. “Atypical” cytology was defined as any case with a cytology report listing the presence of atypical cells with no specific mention as to the presence of adenocarcinoma/malignancy. “Negative” cytology consisted of any case with cytology describing the presence of reactive or benign cells with no specific mention of adenocarcinoma/malignancy or atypia. Any case lacking a cytology diagnosis due to an acellular or poorly preserved specimen was defined as having “indeterminate” cytology.

The degree of cellularity was also defined for each cytology diagnosis and categorized as “sufficient”, “low”, or “non-diagnostic” levels. “Sufficient” cellularity was defined as any case where a cytology diagnosis was made and there was no mention of a scant or paucicellular sample. “Low” level cellularity included cases described as having a scant or paucicellular specimen per the cytology report. “Non-diagnostic” levels included any case where a cytology diagnosis could not be made due to a poor quality specimen per the cytology report.

### Mutational profiling of DNA

For mutational profiling, DNA was extracted from cells microdissected from cytology slides guided by microscopic feature (cellular atypia when present) and from a portion of each cell-free supernatant sample that is normally discarded during preparation of cytology specimens. For the supernatant specimens, DNA was extracted from 5 mL aliquots (Qiagen, Valencia, CA) and then quantified by optical density (NanoDrop, Thermo Scientific, Wilmington, DE). Microdissected cells from cytology slides underwent a similar process for DNA extraction and quantification.

Quantitative PCR was used to establish the amplifiability of DNA from each specimen prior to mutational profiling [22]. Specimens that contained sufficient amounts of amplifiable DNA were categorized as “positive” or “negative” for aggressive disease based on the presence or absence, respectively, of at least one mutation in the form of KRAS point mutation or allelic imbalance (loss of heterozygosity, LOH) mutation in either the supernatant fluid DNA or DNA from microdissected cytology slides. Those with insufficient amounts of amplifiable DNA were categorized as “non-diagnostic” by mutational profiling.

A clinically validated panel was used for mutational profiling [10-15]. The panel included *KRAS* oncogene markers and 16 microsatellite markers (known commercially as *PathFinderTG*, PFTG, RedPath Integrated Pathology, Inc., Pittsburgh, PA). *KRAS* oncogene alterations were examined in codons 12 and 13. The microsatellite markers assessed the presence of allelic imbalance, as measured by loss of heterozygosity (LOH), at 10 genomic loci linked to tumor suppressor genes associated with pancreaticobiliary cancer. These genomic loci (genes) included: 1p (*CMM1, Lmyc*), 3p (*VHL, OGG1*), 5q (*MCC, APC*), 9p (*CDKN2A, CDKN2B*), 10q (*PTEN, MXI1*), 17p (*TP53*), 17q (*NME1, RNF34*), 18q (*DCC*), 21q (*TFF1, PSEN2*), and 22q (*NF2*).

Quantitative allelic imbalance (LOH) and *KRAS* point mutations were determined by PCR and subsequent capillary gel electrophoresis (ABI Genetic Analyzer) [23]. For LOH analysis, the normal allelic balance range was determined to be two standard deviations from the average allelic ratio in which the fluorescence derived from the shorter allele copy is divided by that of the longer allele copy [33]. The presence of LOH was determined by those allelic ratios that fell outside the thresholds. *KRAS* point mutations were established by dideoxy chain termination. Approximations of mutated versus non-mutated (non-neoplastic) DNA for each sample were created by the ratio of wild-type nucleotide and mutant nucleotide peak heights. The mutational profile of microdissected cytology slides and supernatants was characterized as positive for malignancy based on the presence of at least one mutation in any of the 16 LOH or *KRAS* markers.

*KRAS* point mutation analysis was based on dideoxy chain termination (Sanger) sequencing (Applied Biosystems, Grand Island, NY) with a threshold of detection of 8% mutation admixed with 92% wild type DNA. Loss of heterozygosity analysis for detection of genomic deletion was based on fragment analysis using fluorescent labeled PCR primers (Applied Biosystems). Threshold for detection of LOH was 30% mutant DNA admixed with 70% non-mutated DNA for an individual LOH marker. Allelic dropout, a potential technical artifact related to low levels or poor quality (degraded) DNA, was rigorously controlled for by replicate analysis (minimum triplicate) confirming the fidelity of all detectable mutations. Molecular analyses were performed by laboratory personnel blinded to clinical and management features as well as any prior molecular analysis on an individual patient.

## Results

Between August 2011 and May 2012, 39 patients had pancreaticobiliary masses identified by cross-sectional imaging and underwent FNA or biliary brushings. 9/39 patients did not have appropriate follow up information available; therefore, 30 patients were included in the final analysis. Of these 30 patients, 23 underwent FNA and 7 underwent biliary brushing to obtain specimens for cytological and mutational profiling analyses. 21 patients were categorized as having “aggressive” disease, and 9 patients were categorized as having “non-aggressive” disease at the end of the study. In total, 10/30 patients underwent surgery; surgical pathology revealed that 5/10 had adenocarcinoma, 1/10 had intraductal tubulo-papillary neoplasm, and 4/10 had IPMN.

Out of 30 patients, 21 specimens had sufficient cells for cytology diagnosis. Of the 21 cytology specimens with sufficient cellularity, 12 were negative, 5 were atypical, and 4 were positive for aggressive disease. Importantly, 30% of the cytology specimens had either low cellularity (8/30) or a non-diagnostic level of cells (1/30). All 9 specimens were atypical, negative, or indeterminate for aggressive disease; none were positive for malignancy by cytologic diagnosis (Table 1).

**Table 1:** Comparison of cytology diagnoses with degrees of cellularity present in each FNA specimen

**Degree of cellularity**	**Cytology diagnosis (n = 30)**				**Total**
	**Negative**	**Atypical**	**Positive**	**Indeterminate**	
**Sufficient**	12	5	4	0	21
**Low**	4	4	0	0	8
**Non-diagnostic level**	0	0	0	1	1
**Total**	16	9	4	1	30

Table 2 described the disease outcomes of patients compared with the cytology results. 9/30 patients had non-aggressive disease. Of these 9 patients, 7 had corresponding negative cytology diagnoses, 1 had an indeterminate cytology diagnosis and 1 had an atypical cytology diagnosis. 21/30 patients had aggressive disease. Of these 21 patients, 12 had corresponding positive or atypical cytology (Table 2); 4 of these atypical diagnoses were made from a sample that had low cellularity (Table 1). The remaining 9 patients with aggressive disease had corresponding falsely negative cytology (Table 2). 1/9 patients with the false negative cytology had low cellularity (1/4 in Table 1), while the remaining 8/9 patients had sufficient cellularity for a cytology diagnosis (8/12 in Table 1).

**Table 2:** Comparison of patient outcomes with cytology diagnoses

**Cytology diagnosis**	**Patient outcomes (n = 30)**		**Total**
	**Non-aggressive disease**	**Aggressive disease**	
**Negative**	7	9	16
**Atypical**	1	8	9
**Positive**	0	4	4
**Indeterminate**	1	0	1
**Total**	9	21	30

26 patients categorized with a cytology diagnosis of atypical, negative or indeterminate were analyzed by mutational profiling. In 16/26 patients, the supernatant underwent mutational profiling. For 9/26 patients, microdissected cytology slides were tested. And for 1 patient, both the supernatant and microdissected slide were analyzed for mutations. All 26 patients had sufficient quantity and quality of DNA required for mutational profiling. *KRAS* and loss of heterozygosity (LOH) mutations were examined, and 6/17 patients with aggressive disease were positive for *KRAS* mutation. The inclusion of LOH mutations correctly identified an additional 2 cases of aggressive disease for a total of 8/17 cases of aggressive disease identified by mutational profiling. Table 3 describes the presence (positive) or absence (negative) of detectable mutations in microdissected cytology slides or supernatants from patients with non-aggressive disease or aggressive disease with the corresponding cytology diagnoses indicated. For patients with non-aggressive disease, 2 patients, including 1 with atypical and 1 with indeterminate cytology diagnosis, were correctly diagnosed by mutational profiling. All 7 patients with negative cytology were negative by mutational profiling. For patients with aggressive disease, diagnosis by mutational profiling was positive in 7/8 patients with atypical cytology and 1/9 patients with falsely negative cytology. Therefore, mutational profiling was able to provide additional information regarding the presence of aggressive disease in 8/17 patients with either false negative or atypical cytology results.

**Table 3:** Comparison of patient outcomes with negative, not assessable or atypical cytology diagnoses and mutational profiling

**Mutational profiling diagnosis**	**Patient outcome (n = 26)**					
	**Non-aggressive disease**			**Aggressive disease**		
	**Cytology diagnosis**			**Cytology diagnosis**		
	**Atypical**	**Negative**	**Indeterminate**	**Atypical**	**Negative**	**Indeterminate**
**Negative**	1	7	1	1	8	0
**Positive**	0	0	0	7	1	0
**Non-diagnostic**	0	0	0	0	0	0

In our sample of patients, cytology alone detected only 20% (4/21) of aggressive disease with absolute certainty (positive) (Table 2). However, when malignant (positive) cytology testing and the results of mutational profiling on negative, atypical or indeterminate cytology specimens were analyzed together, 57% (12/21) of aggressive cases were detected (Table 4). This increased detection was without a compromise in the ability to conduct first line testing, given that mutational analysis can be performed on discarded or archivable cytology material. The diagnostic yield of specimens was also increased by using the combination of cytology and mutational profiling (100%, 30/30 Table 4) versus cytology alone (97%, 29/30 Table 2). When added to first line cytology testing, mutational profiling of specimens that were atypical, negative, or indeterminate by cytology increased the detection of aggressive disease and the diagnostic yield of specimens regardless of cellularity.

**Table 4:** Comparison of patient outcomes with combined use of malignant (positive) cytology and mutational profiling

**Combined diagnosis**	**Patient outcome (n = 30)**	
**(Cytology & mutational profiling)**	**Non-aggressive disease**	**Aggressive disease**
**Negative**	9	9
**Positive**	0	12
**Non-diagnostic**	0	0

## Discussion

Standard morphologic evaluation of FNA and biliary brushing specimens carries a high false negative rate and at times lacks diagnoses of pancreatic masses and their associated biliary strictures [4-7,34]. This is largely due to sampling variability resulting from FNA and biliary brushing procedures, insufficient amount of cells available within resulting specimens, and difficulty in characterizing cellular atypia in specimens. Our study aimed to better understand the added value of second line mutational profiling when used in conjunction with first line cytology testing to detect aggressive disease when cytology results were inconclusive for the presence of malignancy (i.e. negative, indeterminate or atypical).

The panel of markers used in the mutational profiling for this study was selected based on substantial evidence supporting their involvement in pancreaticobiliary cancers. *KRAS* point mutation has been long-established as a feature of pancreatic cancer [16,35]. Since the presence of LOH is considered a worrisome feature in pancreatic cysts [10,22,36], LOH is an additional logical candidate for mutational profiling of pancreaticobiliary masses. The panel used in this study examined LOH at 10 genomic loci that have been linked to pancreaticobiliary cancer in previous reports [37-39]. Using a combination of these important mutational features, we were able to detect additional cases of aggressive disease beyond that detected by cytology alone in pancreatic masses and their associated biliary strictures.

As reported by others and supported by our study, specimens positive for malignant disease via cytological analysis are very likely to be associated with aggressive disease given the high specificity (95-100%) of cytology for malignancy [40,41]. However, given the poor sensitivity of cytology for detecting aggressive/malignant disease, use of mutational profiling in diagnosing patients was able to detect aggressive disease in an additional 47% (8/17) of the patients, including 1 patient with falsely negative cytology results and 7 patients with atypical cytology results. In addition, no patients with non-aggressive disease had positive results by mutational profiling. Therefore, mutational profiling has the potential to improve diagnostic sensitivity for aggressive disease without compromise to specificity when used as a supplement to first line cytology testing. Such characteristics are of particular utility in cases of low or non-diagnostic cellularity and uncertain cytology diagnoses (i.e. negative, atypical, indeterminate).

Pauci- and acellular specimens can fail to provide sufficient material for cytology as well as cell-based DNA mutational profiling. In these cases, testing of supernatant fluid provides an attractive alternative. Mutational profiling of supernatants may contain higher quality and quantity of DNA and detect more mutations associated with malignancy and in some cases be more representative of the tumor [22] than cytology specimens [24-32]. Our study reinforces the added value of mutational profiling in detecting aggressive disease in both cytology specimens and normally discarded supernatant specimens, with supernatants providing a source of DNA that does not compete with those required by cytology. Mutational profiling of both specimen types can provide additional diagnostic information concerning the presence of aggressive disease, especially when cytology is non-diagnostic/indeterminate or negative, thus, enhancing the early detection of malignancy in patients.

Our study has limitations, including a small sample size that limits our ability to calculate the diagnostic performance of mutational profiling in pancreatic masses and associated biliary strictures. Although mutational profiling allowed us to detect additional cases of aggressive disease, even when cytology and mutational profiling results were combined into one overall diagnosis, 9 cases of malignancy were missed. These falsely negative results are likely due to a combination of the less than perfect sensitivity of both tests as well as to sampling limitations related to FNA and brushing techniques. Despite such limitations, these promising results do provide support for future larger scale studies, with the addition of supernatant analysis providing an opportunity to overcome some of these limitations.

## Conclusion

This study shows that a second line diagnostic test that uses archivable cytology slides and normally discarded supernatant specimens may enhance our ability to detect pancreaticobiliary cancer; when first line cytology results were uncertain (atypical), questionable (negative), or not possible (non-diagnostic/indeterminate), mutational profiling provided additional information regarding the presence of aggressive disease. When used in conjunction with first line cytology testing, mutational profiling increases the number of aggressive cases detected without compromising the specificity associated with cytology results. Using the generally discarded supernatant fluid for analysis gives us the opportunity to perform mutational profiling even when the specimen’s cellularity is at a non-diagnostic level. The recognized presence of false negative and indeterminate first line cytology testing emphasizes the need for second line testing, such as mutational profiling, to improve the early detection of aggressive pancreaticobiliary disease.

## Abbreviations

MP: Mutational profiling; FNA: Fine needle aspirates; LOH: Loss of heterozygosity.

## Competing interests

RedPath Integrated Pathology (RedPath) sponsored this study. Nidhi Malhotra, M.D., Mary Sidawy, M.D., and Nadim Haddad, M.D. received no financial support from RedPath. Sydney Finkelstein, M.D., is a founder of RedPath Integrated Pathology (RedPath). He is the current Chief Scientific Officer and is a shareholder at RedPath. Sara Jackson, Ph.D., Lindsay Freed, B.S., and Mindi Styn, Ph.D., are employees of RedPath. Nidhi Malhotra, M.D., Mary Sidawy, M.D., and Nadim Haddad, M.D. have no competing interests.

## Authors’ contributions

NM, SJ, LF, MAS, and SF participated in the conception/design of study, analysis/interpretation of data, study supervision, drafting of manuscript, and critical revision of manuscript. NH participated in the conception/design of study, analysis/interpretation of data, study supervision, and critical revision of manuscript. MKS participated in the conception/design of study and critical revision of manuscript. NM, SJ, LF, MAS, MKS, NH, and SF read and approved the final manuscript.

## Authors’ information

SF is an anatomical pathologist with special expertise in molecular diagnostics. He is an Adjunct Professor of Pathology at Drexel University.

## Pre-publication history

The pre-publication history for this paper can be accessed here:

http://www.biomedcentral.com/1471-230X/14/135/prepub
